# Quantitative Control of Organ Shape by Combinatorial Gene Activity

**DOI:** 10.1371/journal.pbio.1000538

**Published:** 2010-11-09

**Authors:** Min-Long Cui, Lucy Copsey, Amelia A. Green, J. Andrew Bangham, Enrico Coen

**Affiliations:** 1 Department of Cell and Developmental Biology, John Innes Centre, Norwich, United Kingdom; 2 University of East Anglia, School of Computing Sciences, Norwich, United Kingdom; University of York, United Kingdom

## Abstract

The development of organs with particular shapes, like wings or flowers, depends on regional activity of transcription factors and signalling molecules. However, the mechanisms that link these molecular activities to the morphogenetic events underlying shape are poorly understood. Here we describe a combination of experimental and computational approaches that address this problem, applying them to a group of genes controlling flower shape in the Snapdragon (*Antirrhinum*). Four transcription factors are known to play a key role in the control of floral shape and asymmetry in Snapdragon. We use quantitative shape analysis of mutants for these factors to define principal components underlying flower shape variation. We show that each transcription factor has a specific effect on the shape and size of regions within the flower, shifting the position of the flower in shape space. These shifts are further analysed by generating double mutants and lines that express some of the genes ectopically. By integrating these observations with known gene expression patterns and interactions, we arrive at a combinatorial scheme for how regional effects on shape are genetically controlled. We evaluate our scheme by incorporating the proposed interactions into a generative model, where the developing flower is treated as a material sheet that grows according to how genes modify local polarities and growth rates. The petal shapes generated by the model show a good quantitative match with those observed experimentally for each petal in numerous genotypes, thus validating the hypothesised scheme. This article therefore shows how complex shapes can be accounted for by combinatorial effects of transcription factors on regional growth properties. This finding has implications not only for how shapes develop but also for how they may have evolved through tinkering with transcription factors and their targets.

## Abstract 2

A novel combination of molecular genetics, shape analysis, and computational modelling shows how the complex three-dimensional shape of the Snapdragon flower can arise through local gene activity.

## Author Summary

A major challenge in developmental biology is to understand how patterns of gene activity are translated into complex three-dimensional forms, like hearts, wings, or flowers. Addressing this problem has not been easy, partly because of the difficulties in quantifying the effects of genes on shape and also because we lack frameworks that allow hypotheses about underlying mechanisms to be evaluated. Here we address this issue through a combination of experimental and computational approaches, using the Snapdragon flower as a model system. By quantifying the shapes of these flowers in a range of mutants with reduced or increased activity of particular genes, we show how the complex floral shape depends on the way genes act in combination in each petal region. The proposed interactions were tested by incorporating them into a computational model of the growing flower. Quantitative comparisons reveal a good agreement between the shapes generated by the model and those observed experimentally, confirming our underlying hypothesis. The Snapdragon flower, with its tightly fitting upper and lower petals, has evolved as a specialised mechanism for targeting pollinators. Our article shows how the development and evolution of such forms may have arisen by natural tinkering with the local effects of genes on growth.

## Introduction

Although major progress has been made in the genetic dissection of organ and appendage development, the process whereby gene activities lead to particular tissue shapes is still poorly understood. For example, wing morphogenesis in *Drosophila* is one of the best defined developmental systems [1], yet little is known about how regional gene activities in the imaginal disc are translated into final wing shape [2]. Addressing this problem has not been easy for several reasons. First, genes that modify shape are normally identified through their overall phenotypic effects, making it difficult to establish how particular regions of the tissue are affected. Second, shape is often described in qualitative terms like “rounder” or “more elongated,” making it difficult to quantify and compare the effects of different gene combinations. Third, we lack modelling frameworks that allow hypotheses for how genes control morphogenesis to be evaluated quantitatively.

Here we combine molecular genetic and morphometric approaches to address these issues, using the Snapdragon (*Antirrhinum majus*) flower as a model system. A key advantage of choosing a plant system is that the lack of cell movement means that morphogenesis arises mainly through differential growth. Shape changes can therefore be described in terms of genes modifying rates of growth in particular orientations [3]. So far, this approach has been applied to studying the effects of genes on overall growth rates of an organ [4]. However, it should be possible to extend this principle to the subregions within an organ, thus allowing final shape to be dissected into genetically determined modulations in the local rates and orientations of growth.

**Figure 1:**
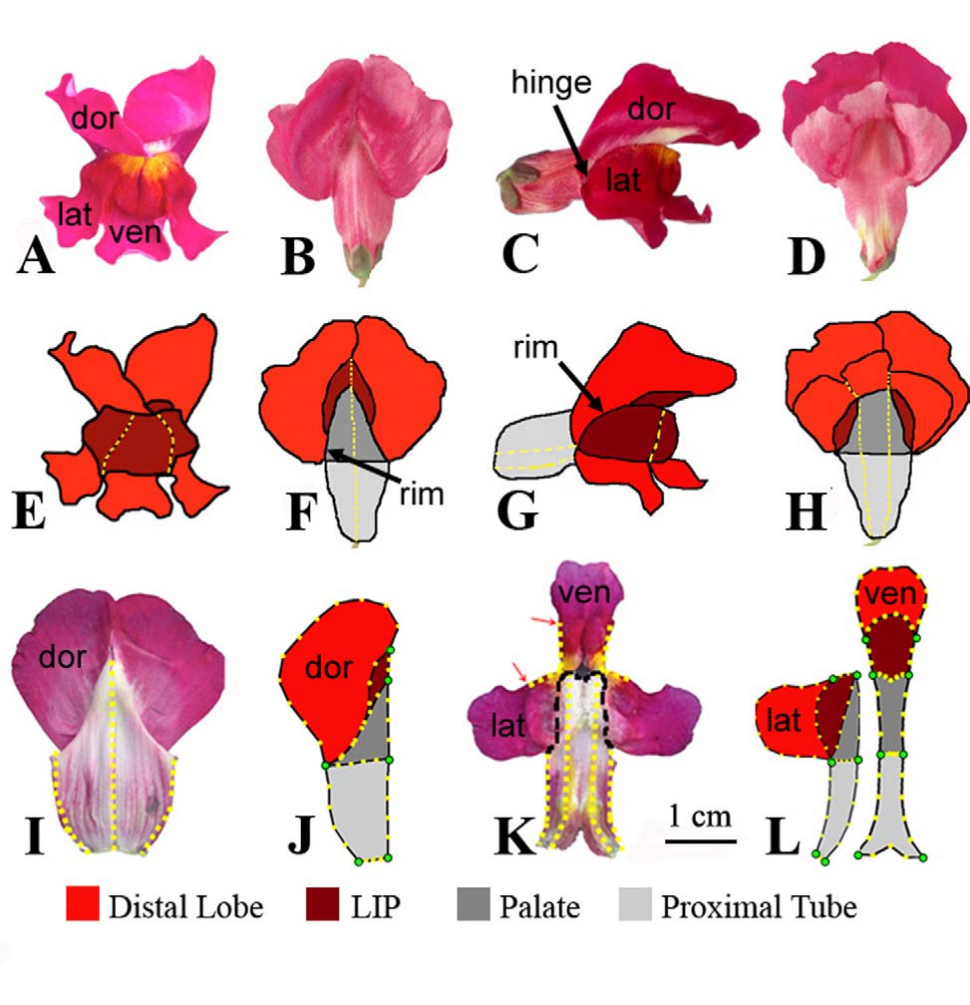
Flower shape of wild-type *Antirrhinum majus*. (A–H) Whole flowers in face view (A,E), dorsal view (B,F), side view (C,G), and ventral view (D,H). (I–L) Flattened petals. Upper corolla section (I) with individual dorsal petal highlighted (J), and lower corolla section (K) with individual lateral and ventral petal highlighted (L). United regions of petals are shown with yellow dotted lines. For (J) and (L) primary landmarks are in green, secondary landmarks in yellow. dor, dorsal; lat, lateral; ven, ventral.

The *Antirrhinum* flower is particularly suitable for this approach as specific shapes can be generated through inactivation or over-expression of key transcription factors. Each flower comprises two upper petals (dorsals) and three lower petals (laterals and ventral) that together form the corolla (Figure 1A–D). The petals are united proximally to form a tube while the distal regions form five lobes. The shapes of the upper and lower petals are precisely matched at the boundary between tube and lobe, termed the rim, so that the overall structure forms a closed mouth hinged at its edges.

The distinctive shapes of the upper and lower petals depend on the activities of four dorsoventral genes: *CYCLOIDEA* (*CYC*), *DICHOTOMA* (*DICH*), *RADIALIS* (*RAD*), and *DIVARICATA* (*DIV*) [5]–[9]. *CYC* and *DICH* encode TCP transcription factors that are expressed from an early stage in the dorsal domain of the flower bud. Mutants lacking both *CYC* and *DICH* have flowers with all petals resembling the ventral petal of wild type. *RAD* and *DIV* encode Myb-like transcription factors. *RAD* is switched on by *CYC* and *DICH* and promotes dorsal identity, while *DIV* is active in lower petals and promotes ventral identity. *DIV* is initially expressed throughout the corolla, but *RAD* is thought to antagonise its activity, preventing *DIV* from acting in dorsal petals. At later developmental stages, *DIV* expression becomes restricted to lateral and ventral petals through the action of the dorsally expressed genes. A *cis*-acting dominant mutant of *CYC* (*backpetals*) has been characterised in which *CYC* is ectopically expressed, leading to lower petals acquiring dorsal identity [9]. However, it is unclear whether the phenotype is a result of ectopic expression of *CYC* and/or its target gene *RAD*.

The changes in shape resulting from inactivation or over-expression of genes may be quantified using morphometric methods. Such methods have been applied previously to genetically controlled shape variations, such as mandible shape in vertebrates, wing shape in *Drosophila*, and leaf shape in plants [10]–[13]. This approach involves placing landmarks at key positions on the organ, aligning the resulting points, and then using multivariate methods to extract major trends in variation. The advantages of taking a quantitative approach are that average shapes for each genotype can be extracted and the main features under genetic control can be highlighted. Additionally, this approach potentially allows quantitative comparisons to be made between experimentally generated shapes and shapes generated by computational modelling, enabling hypotheses about morphogenesis to be evaluated.

Here we show that the genetic control of flower shape can be accounted for by a combination of region-specific effects. We quantify these effects through shape analysis of previously described mutants and of lines in which *RAD* is over-expressed in a range of genetic backgrounds. The shapes observed for multiple genotypes can be summarised with a scheme in which dorsoventral transcription factors act in combination with gene activities along the proximodistal and mediolateral axes to modulate the length or breadth of each petal region. Morphogenetic hypotheses for how these phenotypic effects might arise were evaluated using a modelling framework in which genes modify local polarities and specified growth rates [14],[15]. The petal shapes generated by the resulting model show a good quantitative match with those observed experimentally for each petal from 10 different genotypes, thus validating the underlying hypothesis. Our results suggest that evolution of shape involves a process of “tinkering”, through which size and shape of regions is adjusted by piecemeal modification of local growth properties under the control of transcription factors.

## Results

### Quantifying the Morphology of Wild-Type and Mutant Petals

As a first step towards evaluating the effects of different genes on organ shape, the corolla was subdivided into several regions along its proximodistal axis. Most proximal is a continuous cylinder of tissue, the proximal tube. Beyond this region, the tube tissue extends to form the upper and lower palate (Figure 1E–H). The palate ends distally with a boundary called the rim, which acts as a line of transition between the tube and the lobes. The proximal region of the lobes comprises the lip, over which the lobes of adjacent petals are united (yellow dotted lines in Figure 1E–H). The lip is greatly reduced at the junction between the dorsal and lateral lobes, creating a hinge that allows the corolla to be opened by pollinators. The lobes are separate over the remaining distal region of the lobes.

To quantify the effects of dorsoventral genes on shape, the outline and size of the various regions of the corolla were captured. First, the 3-D structure of the flower was converted into a series of 2-D shapes. To achieve this conversion, the upper and lower sections of the corolla were separated by making cuts along the junction between lateral and ventral petals. The resulting petal sections were then flattened (Figure 1I–L). Second, the outlines of the regions for each petal were captured using a series of landmarks. Eight primary landmarks (green dots in Figure 1J,L) were located at recognisable morphological features, such as where the lobes become separate or where the tube rim and petal junctions intersect. Cell type patterns, which vary along the proximodistal axis of the tube, were also used to define primary landmarks for internal boundaries such as those between ventral and lateral petals. In cases where there were no discernable palate or lip regions, the landmarks bounding these regions were overlaid. The remaining 47 secondary landmarks (yellow dots in Figure 1J,L) were spaced evenly along the outlines of each region between the primary landmarks.

Taken together, the coordinates for the 55 landmarks summarise the shape and size of the regions for each petal. These coordinate values will vary in a correlated manner between petals depending on how the shapes and sizes of the regions are influenced by genotype and petal identity. The main trends or correlations can be captured using Principal Component Analysis (PCA) [16]. To implement this procedure, 110 coordinate values (from 55 landmarks) were determined for dorsal, lateral, and ventral petals from wild type as well as the various genotypes described below. Dorsal, lateral, and ventral petals were sampled from five different flowers for each genotype. Petal shapes were aligned by translation and rotation (Procrustes alignment). The average position for each landmark gave the mean petal shape and region outlines for the population. The major trends of variation about this mean were then determined by PCA on the covariance. This analysis showed that 94% of the variance in coordinate positions could be captured with four principal components (PCs).

**Figure 2:**
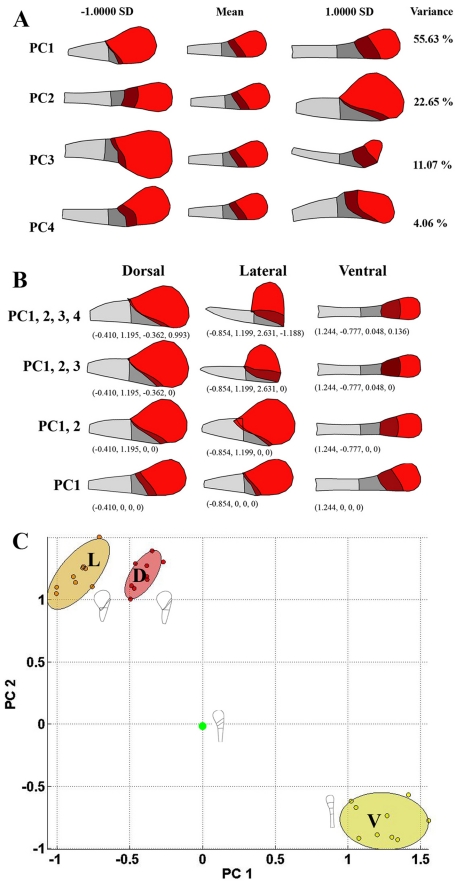
Shape analysis of wild-type petals. (A) Effect on petal shape of varying PCs 1–4. Petal regions colour-coded as in Figure 1. The mean shape, corresponding to all PCs having a value of 0, is shown centrally. The shapes on either side illustrate the effect of increasing or reducing the values of each PC by one standard deviation. (B) Reconstruction of dorsal, lateral, and ventral petals with different numbers of PCs. The values for each PC are shown in brackets. (C) Locations of dorsal (D, red), lateral (L, orange), and ventral (V, yellow) petals in DV space. Position of mean petal shown with green dot. Diagrams show mean shapes for each petal type reconstructed using PC1 and PC2 values. Units for the axes are in standard deviations from the mean.

PC1 accounts for 56% of the variance and captures variation in palate and lip size (Figure 2A). Increasing the value of PC1 gives longer petals with extended lip and palate regions, while reducing PC1 gives shorter petals with a reduced lip and palate. PC2 accounts for 23% of the variance and captures petal asymmetry (Figure 2A). Increasing the value of PC2 gives asymmetric petals with shorter lip and palate regions and a longer distal lobe on one side, while reducing PC2 gives bilaterally symmetrical petals. PC3 accounts for 11% of the variance and captures variation in distal lobe size: increasing the value of PC3 gives a smaller distal lobe, while reducing PC3 gives a larger distal lobe. PC4 accounts for 4% of the variance, with an increase in the PC4 value giving a petal that twists in one direction and a decrease giving a petal twisting the opposite way.

To determine the contribution of each PC to the specification of petal shapes, average PC values for wild-type dorsal, lateral, and ventral petals were determined and then used to reconstruct the petal shapes (Table S1, Figure 2B). If all four PC values were used for reconstruction, the resulting shapes closely resemble the observed shapes (compare top row of Figure 2B with Figure 1J,L). This result is expected because these four PCs capture 94% of the variance in petal shape. A good match was also obtained using just PC1 and PC2, showing that these two PCs are sufficient to capture the main features of the regional shapes. This finding allowed the main shape variations to be represented within a 2-D space that has PC1 and PC2 as its axes. This space will be referred to as the DorsoVentral (DV) space (Figure 2C). Each petal sample corresponds to a point in DV space. The origin of DV space, where all PC values are set to 0, corresponds to the mean petal shape. Samples of the same petal type (e.g., dorsal) form a cloud of points clustered around the mean for that petal type (Figure 2C). The dorsal and lateral clouds are near each other but well separated from the ventral cloud. This clustering reflects the similarity in overall shape and asymmetry of the dorsal and lateral petals and the difference in shape and symmetry of the ventral petals.

### Control of Ventral Petal Development

**Figure 3:**
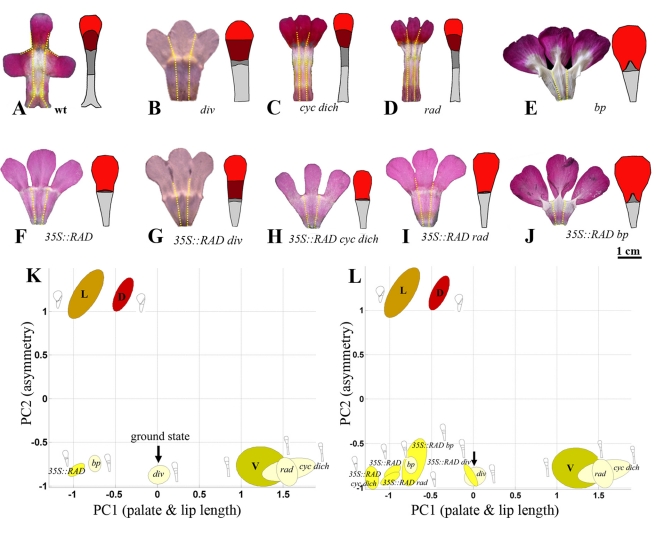
Analysis of ventral petals for various genotypes. (A–J) Flattened lower corolla section with mean ventral petal shape on the right for the wild type (wt) and a series of other genotypes. Petal regions colour-coded as in Figure 1. (K) Positions of ventral petals of single mutants (all pale yellow) and *35S::RAD* (bright yellow) projected onto DV space (from Figure 2C). (L) Positions of ventral petals of *35S::RAD* in various genetic backgrounds (all bright yellow). Arrow points to ground state (*div* ventral petal). Positions of wild-type dorsal (D, red), lateral (L, orange), and ventral (V, dull yellow) petals shown for reference. Diagrams show mean shapes for each petal type reconstructed using PC1 and PC2 values. Units for the axes are in standard deviations from the mean. *bp*, *backpetals*.

To determine the effect of the four dorsoventral genes on the ventrally positioned petal, we analysed its shape in several mutant backgrounds. The only dorsoventral gene expressed in the wild-type ventral petal is *DIV*. The ventral petal of the *div* mutant therefore expresses no dorsoventral genes and can be considered to represent a *ground state*. Relative to the wild-type ventral petal, that of *div* has a reduced palate, is wider, and is not bent back at the rim (Figure 3B). The reduced palate corresponds to a lower value of PC1 (PC1≈0). The *div* mutant is therefore shifted to the left in DV space relative to the wild-type ventral petal (Figure 3K, arrowed). The position of the *div* ventral ground state will be shown in all further DV spaces as a common point of reference. In wild type, expression of *DIV* in the ventral petal throughout development leads to a longer palate and narrower petal than the ground state. Additionally, the wild-type ventral petal bends back at the rim. These observations indicate that *DIV* acts to increase palate length, reduce petal width, and promote bending back at the rim.

*CYC*, *DICH*, and *RAD* are not expressed in the lower corolla section, so we would not expect these genes to have much effect on ventral petal shape. Consistent with this expectation, the shapes of the *cyc dich* and *rad* mutant ventral petals are similar to wild type (Figure 3C,D) and map to similar positions in DV space (Figure 3K). In contrast, the ventral petal of *backpetals* is markedly different from wild type, showing a reduced lip (Figure 3E). The reduced lip size correlates with a leftward shift in DV space (Figure 3K). Additionally, the distal lobe region of *backpetals* is larger than wild type, particularly along its lateral edges (giving a low value of PC3; Table S1). Also, similar to the ground state, the ventral lobe does not bend back at the rim in *backpetals*. *Backpetals* is a semidominant *CYC* allele that expresses *CYC* and its downstream target *RAD* ectopically in the ventral and lateral petals [9]. The effect of *backpetals* on ventral petal shape may therefore reflect the action of *CYC* or *RAD* or the combined action of both genes.

**Figure 4:**
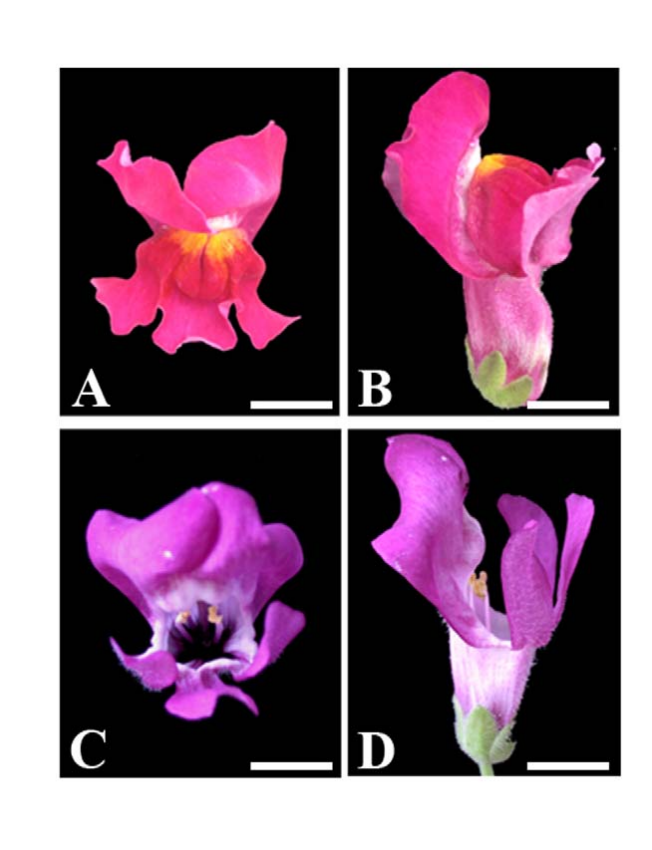
Effect of ectopic *RAD* expression in *Antirrhinum majus*. Comparison between wild-type (A,B) and *35S::RAD* (C,D) flowers. Face views on left (A,C), side views on right (B,D). Scale bar  = 1 cm.

To separate the contributions of *CYC* and *RAD*, we generated plants that expressed *RAD* ectopically, by introducing *RAD* under the control of the 35S promoter. The ventral petals from these transgenic plants should express *RAD* but not *CYC*. Three transgenics were obtained, two of which showed strong petal phenotypes (Figure 4). No phenotypic effects were observed in leaves, even though *RAD* expression was detected by RT-PCR of the transgenics but not in wild type (unpublished data).

The most noticeable effect of ectopically expressing *RAD* in the ventral petal was reduction of both the lip and palate regions (Figures 4 and 3F). This reduction resulted in the *35S::RAD* point cloud mapping to a similar position to *backpetals* in DV space (with a low value of PC1) (Figure 3K). Also, like *backpetals*, the *35S::RAD* ventral petal lobe does not bend back. Thus, *RAD* can exert an autonomous effect on petal shape in the absence of *CYC*. However, the phenotype of *35S::RAD* is not identical to that of *backpetals*. Unlike *35S::RAD*, *backpetals* has a slightly enlarged medial palate and a large distal lobe (compare Figure 3E,F), indicating that *CYC* acts partly independently of *RAD* to increase the length of these regions.

To explore interactions between the dorsoventral genes further, *35S::RAD* was introduced into several mutant backgrounds (Figure 3G–J). Analysis of the ventral petals showed that the tube of *35S::RAD div* resembled that of the *div* ground state, having a reduced palate (compare Figure 3B with Figure 3G). This result is consistent with previous proposals that a major effect of *RAD* is to antagonise *DIV* [7],[8]. Additionally, the *35S::RAD* ventral lip is greatly reduced compared to *div*, and the palate is also further reduced (the PC1 value for *35S::RAD* is much less than for *div*). This finding indicates that *RAD* acts independently of *DIV* to reduce lip and palate length. The phenotype of *35S::RAD* in ventral petals resembles that of *35S::RAD rad* and *35S::RAD cyc dich*. This result is expected because *RAD*, *CYC*, and *DICH* are not normally expressed in ventral petals. In a *backpetals* mutant background, *35S::RAD* had little effect on ventral petal shape, also expected as *RAD* is already expressed ectopically in the *backpetals* mutant.

### Control of Dorsal Petal Development

**Figure 5:**
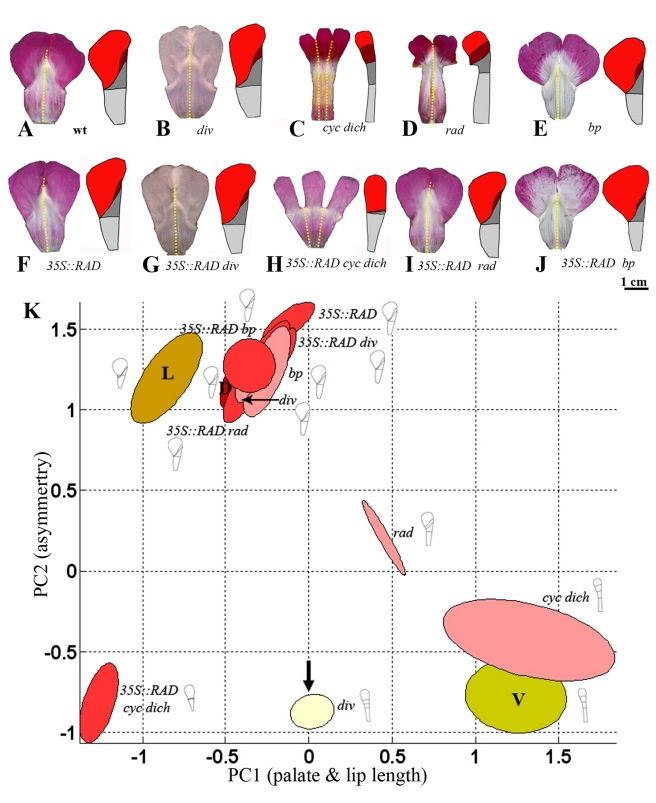
Analysis of dorsal petals for various genotypes. (A–J) Flattened upper corolla sections with mean dorsal petal shape to the right for wild type (wt) and a series of other genotypes. Petal regions colour-coded as in Figure 1. (K) Positions of dorsal petals of various genotypes (colour-coded red or pink) projected onto DV space. Arrow points to ground state (*div* ventral petal, pale yellow). Positions of wild-type dorsal (D, red), lateral (L, orange), and ventral (V, dull yellow) shown for reference. Diagrams show mean shapes for each petal type reconstructed using PC1 and PC2 values. Units for the axes are in standard deviations from the mean. *bp*, *backpetals*.

We next analysed the effect of dorsoventral genes on dorsally positioned petals (Figure 5). Wild-type dorsal petals express *CYC*, *DICH*, and *RAD* and also *DIV* at early stages. The main difference between wild-type dorsal petals and the ground state is the increased value of PC2, reflecting a marked asymmetry in petal shape. This asymmetry involves a reduced lip on one (lateral) side of the petal and an extended palate on the other (dorsal) side (Figure 5A). Extension of the palate on the dorsal side of the petal most probably reflects *DICH* activity, as palate asymmetry is not observed in the ventral petal of *backpetals* (Figure 3E), which only differs from wild-type dorsal petals in not expressing *DICH*. Reduction of length on the lateral side of the wild-type dorsal petal depends on *RAD* activity. In the *rad* mutant, lip length is restored to this side, reducing the degree of petal asymmetry (Figure 5D). The *rad* dorsal petals remain asymmetric because *DICH* activity increases palate length on the more dorsal side.

In *cyc dich* mutants the dorsally positioned petals are fully ventralised (Figure 5C). The petals are bilaterally symmetric because they lack both *DICH* and *RAD* expression (activation of *RAD* depends on *CYC* and *DICH*). The absence of *RAD* also leads to ectopic *DIV* activity in *cyc dich* dorsal petals (*RAD* normally antagonises *DIV*), accounting for the extended palate and higher value of PC1 relative to the ground state (Figure 5K). If *RAD* is ectopically expressed in *cyc dich* dorsal petals (*35S::RAD cyc dich*), the PC1 value drops below that of the ground state, as lip and palate regions both become reduced (Figure 5H). This result is consistent with *RAD* reducing lip and palate length and also further reducing palate length by antagonising *DIV*.

The *div* mutation does not affect dorsal petal development (Figure 5B), presumably because *DIV* activity is normally blocked in dorsal petals by expression of *RAD*. Dorsal petal development is also not affected by the *backpetals* mutation (Figure 5E), as expected because *backpetals* does not modify gene expression in the dorsal domain. *35S::RAD* also had little or no effect on dorsal petals in wild-type, *div*, or *backpetals* backgrounds (Figure 5G,J). Again this result was expected because the endogenous *RAD* gene is expressed in dorsal petals. *35S::RAD rad* dorsal petals have a wild-type phenotype, showing that the transgene complements *rad* in dorsal regions. This result demonstrates that the shape of the wild-type dorsal petal does not depend on spatial regulation of *RAD* expression within the dorsal petal.

### Control of Lateral Petal Development

**Figure 6:**
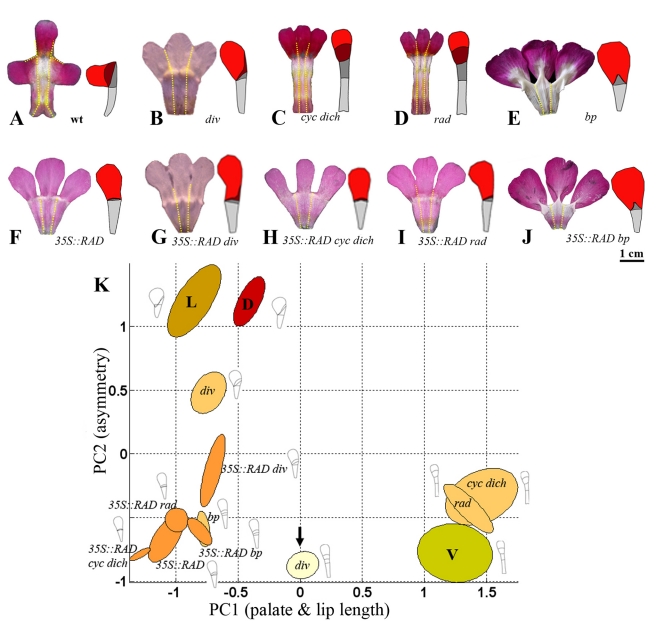
Analysis of lateral petals for various genotypes. (A–J) Flattened upper corolla sections with mean lateral petal shape for wild type (wt) and a series of other genotypes. Petal regions colour-coded as in Figure 1. (K) Positions of lateral petals of various genotypes (colour-coded orange) projected onto DV space. Arrow points to ground state (*div* ventral petal, pale yellow). Positions of wild-type dorsal (D, red), lateral (L, orange), and ventral (V, dull yellow) shown for reference. Diagrams show mean shapes for each petal type reconstructed using PC1 and PC2 values. Units for the axes are in standard deviations from the mean. *bp*, *backpetals*.

We next analysed laterally positioned petals in various genetic backgrounds. Similar to the wild-type dorsal petal, each wild-type lateral petal is asymmetric with a reduced lip and palate on one (lateral) side and extended lip and palate on its other (ventral) side (Figure 6A). This morphology places lateral petals in a similar position to dorsal petals in DV space. However, in lateral petals asymmetry of the palate depends on *DIV* rather than *DICH*. In the *div* mutant, the palate is shortened on its ventral side, leading to a more symmetric shape (lower PC2 value, Figure 6B,K). The *div* lateral petals are still asymmetric because lip and palate length is reduced on the more lateral side of the petal. This reduction involves *RAD*. In *rad* mutants, the lateral petal becomes bilaterally symmetrical, with extended lip and palate regions (Figure 6D). The extended palate mainly reflects ectopic *DIV* activity (*DIV* is no longer antagonised by *RAD*), while the extended lip reflects lack of *RAD* activity. As *RAD* is not normally expressed in the lateral domain, the reduction of lateral lip growth in wild-type lateral petals involves a non-autonomous effect of *RAD* expression from the adjacent dorsal domain. If *RAD* is expressed ectopically in the lateral petal, as in *35S::RAD* genotypes, the length of the lip and palate regions becomes negligible and the petal bilaterally symmetrical, with a low PC2 value, similar to that of the ground state (Figure 6F). The value of PC1 value drops below the ground state, reflecting *RAD* antagonising *DIV* and also reducing lip length (Figure 6K). Lateral petals of *35S::RAD backpetals* are bilaterally symmetrical, like *35S::RAD*, but have a partially extended medial palate (Figure 6J). This suggests that expressing *CYC* counteracts the effect of *RAD* on reducing palate length in medial regions.

## Discussion

**Figure 7:**
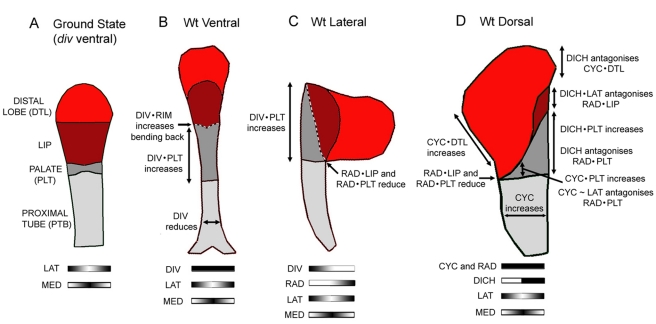
Combinatorial effects of dorsoventral genes. In the following summaries of gene interactions, a dot (•) indicates “in combination with” while a tilda (∼) indicates “in the absence of.” (A) Ground state (*div* ventral petal). The basic petal shape is determined by gene activities that vary along the proximodistal (PTB, PLT, LIP, and DTL) and mediolateral (MED and LAT) axes. (B) Wild-type ventral petal. DIV is expressed throughout the petal. DIV reduces petal width while DIV•PLT increases palate length. DIV•RIM promotes bending back of the lobe (dotted line). (C) Wild-type lateral petal. Non-autonomous RAD activity from the dorsal side restricts DIV activity towards the ventral side at later stages of development. RAD•LIP and RAD•PLT reduce palate and lip length on one side while DIV•PLT increases palate length on the other. The lobe is bent back by DIV•RIM (at early stages, when DIV is expressed throughout the petal). (D) Wild-type dorsal petal. CYC and RAD are expressed throughout while DICH is expressed in the most dorsal half. CYC increases petal width. CYC•PLT and DICH•PLT increase palate length, while reduction in length by RAD•PLT is antagonised by DICH and CYC∼LAT. Reduction in lip length by RAD•LIP is antagonised by DICH•LAT, leading to a visible lip on the dorsal side. CYC•DTL increases length of the distal lobe, which is antagonised by DICH on the dorsal side.

Analysis of petal phenotypes in wild-type, mutant, and transgenic backgrounds reveals that the dorsoventral genes have several region-specific effects on shape. These effects on local shape can be accounted for by a scheme in which the dorsoventral genes interact combinatorially with a pattern of gene activities along the proximodistal and mediolateral axes (Figure 7). Candidate genes for the proximodistal gene activities are the *LIP1* and *LIP2* genes, which encode AP2-like transcription factors that increase palate and lip length [17], and *CIN*, which encodes a TCP transcription factor that increases lip length [18]. These genes may play an equivalent role to proximodistal systems involved in animal limb development [19]. Less is known about mediolateral systems in plants [20], although a notable feature in our scheme is that it involves graded changes, allowing lengths to be increased or decreased smoothly. This pattern may be similar to the way graded mediolateral information is provided by Dpp during *Drosophila* wing development [21],[22]. The scheme also involves graded effects for *RAD* activity, which spreads non-autonomously from the dorsal into the lateral domain to restrict *DIV* function. This spread may reflect direct movement of the RAD protein, as described for other small plant Myb proteins [23], or more indirect spreading mediated by signalling molecules.

**Figure 8:**
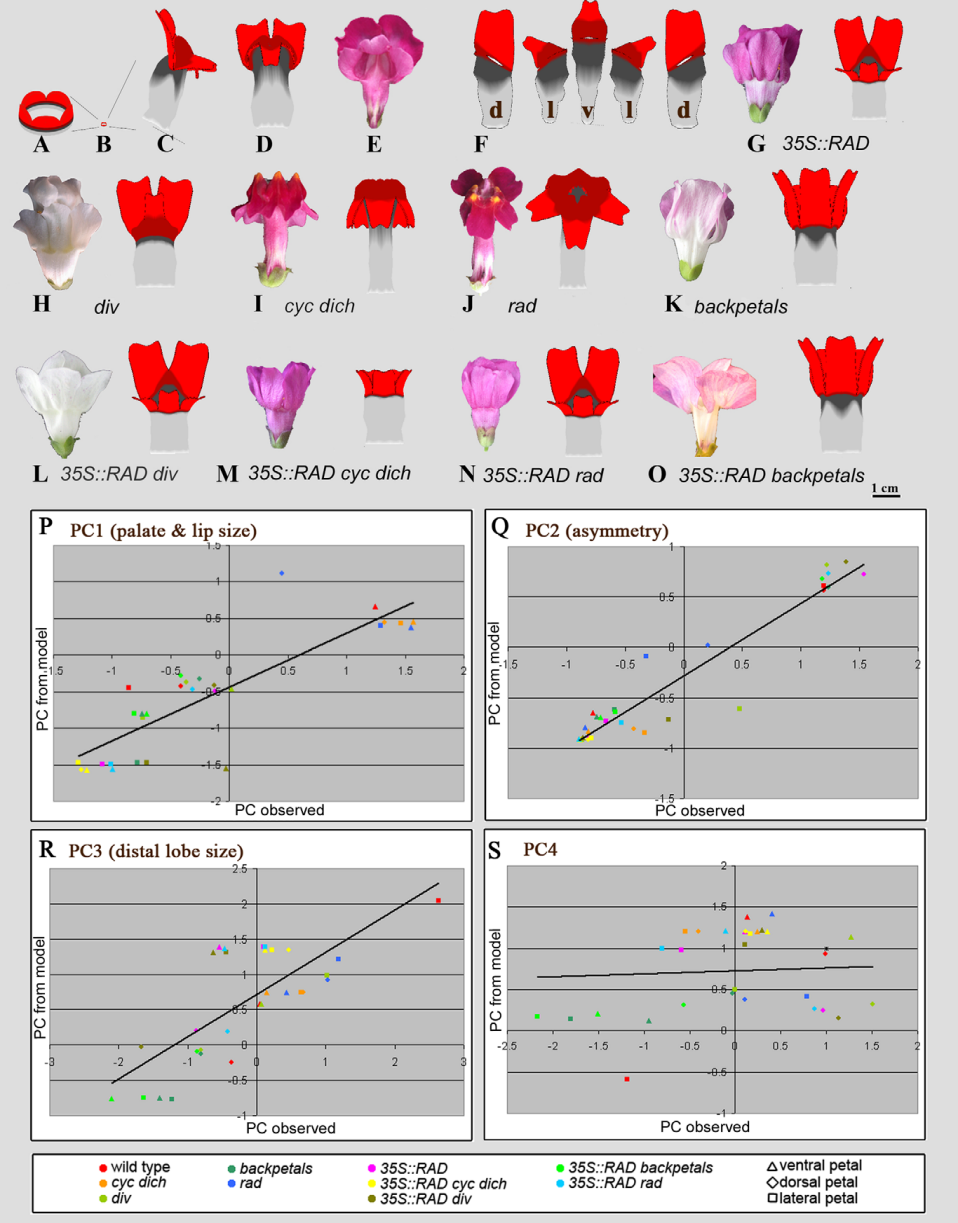
Comparison of observed corolla shapes with growth model corolla shapes. (A) Model corolla at initial developmental stage. (B) Initial model stage at same scale as (C). (C) Side view of wild-type corolla generated by growth model. (D) Ventral view of wild-type flower generated by growth model. (E) Ventral view of real flower. (F) Computationally flattened dorsal (d), lateral (l), and ventral (v) petals from the growth model. Petal regions colour-coded as in Figure 1. (G–O) Ventral view of mutants described in this article, with real flower on left and result from growth model on right. (P–S) Correlation between PC values for petals of observed and modelled petals. Each point represents the PC value obtained from the model for a particular petal type and genotype, plotted against the observed mean PC value for the corresponding petal and genotype.

Although the scheme in Figure 7 can account for the observed phenotypes through combinatorial effects on the shape and size of regions, it does not define the morphogenetic processes through which shapes are generated. To generate phenotypic outcomes, such as an increase or decrease in length of a petal region, genes presumably modify rates of growth along particular orientations within the region as it develops. However, predicting the consequences of particular hypotheses for growth control can be difficult for several reasons. One is that local orientations may become deformed through differential growth, dynamically modifying the principal orientations in which a region grows. Secondly, the extent to which a region grows may be mechanically constrained by neighbouring regions; so specified growth need not be the same as resultant growth. To address these issues, a computational modelling approach for growing tissues, called the GPT-framework (Growing Polarised Tissue framework), was used to determine the consequences of particular hypotheses [24]. The petal was modelled as a growing material sheet of tissue that can deform in 3-D, incorporating the combinatorial interactions described in Figure 7 [14]. Dorsoventral genes such as CYC and DICH were assumed to be expressed uniformly throughout development within their domains. According to the GPT-framework, genes influence shape by modifying tissue polarity and specified rates of growth (rates of extension along axes defined by the local polarity). For example, the combination DIV·PAL increases palate length by promoting specified growth parallel to the local polarity. Tissue polarity is established through three organisers (proximal, central, and distal), from which polarity signals propagate through the tissue. The activity of these organisers is also influenced by dorsoventral genes [14]. Figure 8 shows the output from the growth model for wild type, from the starting shape of a small lobed cylinder of tissue (Figure 8A,B) through to the final shape (Figure 8C,D).

To test the hypotheses underlying the computer model, the various genotypes described in this article were generated by setting the relevant gene activity in the model to 0 (null mutants) or to 1 everywhere (over-expression lines). The resulting corollas showed a good qualitative match to observed flowers (Figure 8G–O). To give a more quantitative comparison, petals from each model corolla were computationally flattened (e.g., Figure 8F) and their outlines processed in the same way as the observed petal data. The PC values from the model were then compared to the PC values observed experimentally for the corresponding genotype and petal (Table S1; Figure 8P–S). As can be seen in Figure 8P,Q, there is a strong correlation between model output and observational data for PC1 (*R* ^2^ = 0.87, *p*<0.0001) and PC2 (*R* ^2^ = 0.91, *p*<0.0001). This result shows that the model captures the main relationships between genes and shape for each petal and thus provides quantitative validation of the proposed combinatorial interactions between the dorsoventral genes proposed in Figure 7. Values for PC3 also show a significant correlation between observed and modelled (*R* ^2^ = 0.56, *p*<0.0001; Figure 8R), suggesting that the model also captures this aspect of petal shape variation. However, PC4 showed little correlation (*R* ^2^ = 0.04, *p* = 0.28; Figure 8S), which is not surprising because this PC captures only minor shape variations.

In the growth model, each dorsoventral gene has several region-specific effects on rates or orientations of growth. This hypothesis is consistent with these genes encoding transcription factors that act in combination with other factors to influence a variety of target genes. These interactions may have been elaborated during the evolution of the *Antirrhinum* lineage, leading to the formation of a corolla with a closed mouth, hinged at its edges. Such evolutionary tinkering [25] would have included promotion of dorsal and ventral palate growth, by *DICH* and *DIV*, respectively, repression of lip growth at the lateral petal boundaries by *RAD* to create a hinge, and promotion of tissue polarity organisers at particular locations. Thus, the close match between upper and lower petals depends on a history of multiple regional modifications. Similar principles may underlie the close match between the upper and lower jaws of vertebrates, illustrated by mutants in which the lower jaw protrudes or recedes [26]–[28]. The evolution of matched tissue shapes can be compared to the way protein domains may evolve to match each other [29]. In both cases shape-matching arises through tinkering, involving either a sequence of adjustments in regional growth properties and polarities as described here or a series of modifications to protein shape through piecemeal amino acid changes.

## Materials and Methods

### *Antirrhinum majus* Stocks

Plants of JI 7 (wild type), JI 98 (wild type), JI 726 (*rad*-726), JI 609 (*rad-*609), JI 721 (*cyc-721*), JI 608 (*cyc*-608), JI 705 (*backpetals-705*), JI 13 (*div*-35 [5]), and JI 718 (*cyc-608 dich-719*) were grown in the greenhouse as described previously [30] and recurrently crossed with 35S::RAD transgenic *Antirrhinum majus* lines. Stocks JI 7 and JI 98 were used as the standard wild type for comparison with the mutants.

### *Antirrhinum majus* Transformation

The 35S::RAD construct was cut from a pGREEN0029 [31] vector and transformed into a binary vector pBIN 19 [32]. This expression vector was transformed into *Agrobacterium* strain GV3101 and used to transform *Antirrhium majus* as described by [33]. Three kanamycin resistant shoots were obtained and analysed by PCR using a set of primers for the kanamycin resistance gene (Neomycin phosphotransferase II), 5′-GATGGATTGCACGCAGGTTC-3′ and 5′-GTGGTCGAATG GGC AGGTAG-3′. A strong phenotype 35S::RAD transgenic line and a weak phenotype 35S::RAD transgenic line were crossed with each of the mutants listed above.

### Genotyping

The back-crossed plants were screened on MS medium containing 50 mg/l kanamycin and genotyped using the primer sets described below. Genotyping of mutant alleles was performed by PCR using combinations of gene-specific or transposon-specific primers. Primers were 5′-aggttttatgcgacgaattttg3′ and 5′-aggttttatgcgacgaattttg-3′ for *rad*-726; 5′-atgtttgggaagaacacata-3′ and 5′-ctaattgatgaacttgtgct-3′ for *cyc*-721; 5′aggttctgactatctgcgcc-3′ and 5′-gtccagtcctttgtcacgtg-3′ for *backpetals-705*; 5′-atggcttcgactcgtggttc-3′ and 5′-taaggaagcttcgggtccgg-3′ for *rad*-609; 5′-atgtttgggaagaacacata-3′ and 5′-gtgacccatgcactcttgg-3′ for *cyc-608*; and 5′-gggtgttccttggacagaag-3′ and 5′-tcatgcgttcggaaagtgaag-3′ for *div-35*. The *div* mutant allele was detected by sequencing PCR products.

### Analysis of Expression

To detect *RAD* and transgene expression, total RNA was extracted from young leaves using an RNeasy Plant Mini Kit (Qiagen, UK). First-strand cDNA was synthesised using the SuperScript III First-Strand Synthesis System for RT-PCR (Invitrogen) on 5 µg of total RNA treated with a TURBO DNA-free kit (Ambion). RT-PCR was carried out using specific primer sets: 5′-atggcttcgactcgtggttct-3′ and 5′-gaattttgagatttctgaacc-3′ for *RAD* expression; 5′-agatggattgcacgcaggttc-3′ and 5′-gtggtcgaatgggcaggtag-3′ for *NPT*II expression; and 5′-attggtgctgaggttgaga-3′ and 5′-acaactgactccagcaaacg-3′ for ubiquitin expression. PCR was performed for 4 min at 94°C and then 30 cycles consisting of 40 s at 94°C, 40 s at 61°C and 60 s at 72°C, followed by 10 min at 72°C.

### Shape Model Analysis

Flower samples were collected from eight individual plants each from mutant and transgenic lines, when flowers were fully opened. Each flower was dissected by cutting in a proximodistal direction along the tube conjunction of dorsal and lateral petals, using a razor. The upper petals (including two dorsal petals) and lower petals (including two lateral petals and a ventral petal) were flattened by gluing onto paper and photographed using a Nikon Coolpix 995 digital camera. All images were normalised to 4000 pixels/cm^2^ using an ImagePrep tool written in Matlab. Fifty-five landmarks (eight primary landmarks and 47 secondary landmarks) were fitted to each of the dorsal, lateral, and ventral petals (Figure 1J,L) to build the shape model using the AAMToolbox (http://fizz.cmp.uea.ac.uk/wiki/DArT_Toolshed/index.php/Main_Page) in Matlab (version: 7.2), as described in [12]. A statistical PCA model of flower petal shape and size was generated from the petal point models of the mutant and transgenic plant dataset, projected to a morphospace defined by PC1 and PC2.

## Supporting Information

Table S1 **Principal Component values for petals of various genotypes compared to values obtained from petals generated with the growth model.** DP, dorsal petal; LP, lateral petal; VP, ventral petal.
(0.02 MB XLS)

## Abbreviations

D: dorsal (petal)
DV: dorsoventral
GPT-framework: Growing Polarised Tissue framework
L: lateral (petal)
PC: principal component
PCA: principal component analysis
V: ventral (petal)

## References

[1] Held L. I (2002). Imaginal discs: the genetic and cellular logic of pattern formation:.

[2] Dworkin I, Gibson G (2006). Epidermal growth factor receptor and transforming growth factor-beta signaling contributes to variation for wing shape in Drosophila melanogaster.. Genetics.

[3] Hejnowicz Z, Romberger J. A (1984). Growth tensor of plant organs.. Journal of Theoretical Biology.

[4] Sinnott E (1960). Plant morphogenesis:.

[5] Galego L, Almeida J (2002). Role of *DIVARICATA* in the control of dorsoventral asymmetry in *Antirrhinum* flowers.. Genes and Development.

[6] Almeida J, Rocheta M, Galego L (1997). Genetic control of flower shape in *Antirrhinum majus*.. Development.

[7] Corley S. B, Carpenter R, Copsey L, Coen E (2005). Floral asymmetry involves an interplay between TCP and MYB transcription factors in *Antirrhinum*.. PNAS.

[8] Costa M. M. R, Fox S, Hanna A. I, Baxter C, Coen E (2005). Evolution of regulatory interactions controlling floral asymmetry.. Development.

[9] Luo D, Carpenter R, Copsey L, Vincent C, Clark J (1999). Control of organ asymmetry in flowers of *Antirrhinum*.. Cell.

[10] Zimmerman E, Palsson A, Gibson G (2000). Quantitative trait loci affecting components of wing shape in Drosophila melanogaster.. Genetics.

[11] Klingenberg C. P, Leamy L. J (2001). Quantitative genetics of geometric shape in the mouse mandible.. Evolution.

[12] Langlade N. B, Feng X, Dransfield T, Copsey L, Hanna A. I (2005). Evolution through genetically controlled allometry space.. PNAS.

[13] Klingenberg C. P (2010). Evolution and development of shape: integrating quantitative approaches.. Nat Rev Genet.

[14] Green A. A, Kennaway J. R, Hanna A. I, Bangham J. A, Coen E (2010). Genetic Control of Organ Shape and Tissue Polarity.. PLoS Biol.

[15] Kennaway J, Coen E, Green A, Bangham A (2010). Generation of diverse biological forms through combinatorial interactions between tissue polarity and growth.. Manuscript submitted.

[16] Matthews I, Cootes T. F, Bangham J. A, Cox S, Harvey R (2002). Extraction of visual features for lip reading.. IEEE PAMI.

[17] Keck E, McSteen P, Carpenter R, Coen E (2003). Separation of genetic functions controlling organ identity in flowers.. Embo Journal.

[18] Crawford B. C, Nath U, Carpenter R, Coen E. S (2004). *CINCINNATA* controls both cell differentiation and growth in petal lobes and leaves of *Antirrhinum*.. Plant Physiol.

[19] Pueyo J. I, Couso J. P (2005). Parallels between the proximal-distal development of vertebrate and arthropod appendages: homology without an ancestor?. Curr Opin Genet Dev.

[20] Byrne M. E (2005). Networks in leaf development.. Curr Opin Plant Biol.

[21] Teleman A. A, Cohen S. M (2000). Dpp gradient formation in the Drosophila wing imaginal disc.. Cell.

[22] de Navas L. F, Garaulet D. L, Sanchez-Herrero E (2006). The ultrabithorax Hox gene of Drosophila controls haltere size by regulating the Dpp pathway.. Development.

[23] Kurata T, Ishida T, Kawabata-Awai C, Noguchi M, Hattori S (2005). Cell-to-cell movement of the CAPRICE protein in Arabidopsis root epidermal cell differentiation.. Development.

[24] Kennaway J, Green A, Coen E, Bangham A (submitted) Generation of diverse biological forms through combinatorial interactions between tissue polarity and growth.

[25] Jacob F (1977). Evolution and tinkering.. Science.

[26] Hide T, Hatakeyama J, Kimura-Yoshida C, Tian E, Takeda N (2002). Genetic modifiers of otocephalic phenotypes in Otx2 heterozygous mutant mice.. Development.

[27] Schilling T. F, Walker C, Kimmel C. B (1996). The chinless mutation and neural crest cell interactions in zebrafish jaw development.. Development.

[28] Wilkie A. O, Morriss-Kay G. M (2001). Genetics of craniofacial development and malformation.. Nat Rev Genet.

[29] Pazos F, Valencia A (2008). Protein co-evolution, co-adaptation and interactions.. EMBO J.

[30] Carpenter R, Martin C, Coen E. S (1987). Comparison of genetic behavior of the transposable element Tam3 at 2 unlinked pigment loci in antirrhinum-majus.. Molecular & General Genetics.

[31] Baxter C. E, Costa M. M, Coen E. S (2007). Diversification and co-option of RAD-like genes in the evolution of floral asymmetry.. Plant J.

[32] Bevan M (1984). Binary Agrobacterium vectors for plant transformation.. Nucleic Acids Res.

[33] Cui M. L, Handa T, Ezura H (2003). An improved protocol for Agrobacterium-mediated transformation of Antirrhinum majus L.. Mol Genet Genomics.

